# A Novel Zirconium Modified Arylacetylene Resin: Preparation, Thermal Properties and Ceramifiable Mechanism

**DOI:** 10.3390/polym12030684

**Published:** 2020-03-19

**Authors:** Qilin Mei, Honghua Wang, Xiaocheng Chen, Ying Wang, Zhixiong Huang

**Affiliations:** School of Materials Science and Engineering, Wuhan University of Technology, 122 Luoshi Road, Wuhan 430070, China; meiqilin@whut.edu.cn (Q.M.); 13343434177@163.com (H.W.); cking2008@126.com (X.C.); 374143382@163.com (Y.W.)

**Keywords:** arylacetylene, zirconium, thermal stability, ceramifiable mechanism

## Abstract

With the rapid development of thermal protection systems for the aerospace industry and power electronics, polyarylacetylene (PAA) resin plays an important role because of its good mechanical properties, high glass transition temperature (T_g_), low water absorption, high char yield (Y_c_), and the fact that there is no byproduct released in the curing process. In order to further improve the thermal property of PAA based FRP for the thermal protection field, the introduction of a zirconium element into arylacetylene is promising. In this paper, zirconium modified arylacetylene (ZAA) resin was prepared by two-step synthesis. The FTIR analysis characterized its molecular structure and confirmed the products. The viscosity of ZAA was about 6.5 Pa·s when the temperature was above 120 °C. The DSC analysis showed that the ZAA had a low curing temperature, and its apparent activation energy was 103.86 kJ/mol in the Kissinger method and 106.46 kJ/mol in the Ozawa method. The dielectric constant at 1 MHz of poly(zirconium modified arylacetylene) (PZAA) was 3.4. The TG analysis showed that the temperatures of a weight loss of 5% (T_d5_) and char yield (Y_c_) at 800 °C of PZAA were 407.5 °C and 61.4%, respectively. The XRD results showed the presence of SiO_2_ and ZrO_2_ in the PZAA residue after ablation. The XRF results showed that the contents of SiO_2_ and ZrO_2_ in PZAA residual after ablation were, respectively, 15.3% and 12.4%. The SEM showed that the surface of PZAA after ablation had been covered with a dense and rigid ceramic phase composed of ZrO_2_ and SiO_2_. Therefore, the introduction of Zr into arylacetylene greatly improved the densification of the surface after ablation, and improved the heat resistant property.

## 1. Introduction

With the rapid development of thermal protection systems for the aerospace industry and power electronics, high thermally stable fiber reinforced polymer composites (FRP) have been playing important roles because of their low density, high temperature resistance, high strength and high modulus [1,2,3,4]. The thermal property of FRP is mainly determined by the matrix resin. The matrix resin must maintain a reliable structure and provide a good mechanical property in high temperature. As it possesses a high crosslinking degree and a high carbon residue rate, polyarylacetylene (PAA) resin is used as a resin matrix for high thermally stable FRP [5,6,7]. Compared with traditional phenolic resins applied in thermal protection systems, PAA has excellent properties, such as good mechanical properties, a high glass transition temperature (T_g_), low water absorption, high char yield (Y_c_) and the fact that there is no byproduct released in the curing process. The curing mechanism of arylacetylene mainly contains a Dielse Alder addition, the ring trimerization and radical polymerization. Nevertheless, the thermal stability of PAA is not enough to meet the high requirements for materials in aerospace and power electronics. As a result, there is a need to further enhance the thermal stability and widen its application.

At present, the arylacetylene polyimide resin and silicon-containing arylacetylene resin have been played much emphasis. Therein, arylacetylene polyimide resin was studied in many research papers, and the obtained resins had good processing properties, thermal stability, thermal oxidation resistance properties and mechanical properties [8,9,10]. The introduction of a silicon element into the molecular structure of arylacetylene, mainly modified organic silicon, can greatly improve the thermally stable performance [11,12,13,14,15]. Additionally, the comprehensive properties have been improved in the silicon modified PAA, such as the deceased brittleness, improved toughness and mechanical properties and extra ceramifiable properties. Li Q. et al. [16] synthesized a novel arylacetylene oligomer containing polyhedral oligomeric silsesquioxane (POSS) in main chains. The results showed that the temperatures of a weight loss of 5% (T_d5_) could reach 503 °C and Y_c_ in nitrogen at 800 °C could reach 87.1%. Guo K. K. et al. [17] studied the thermal curing, thermal property and degradation behavior of silicon-containing arylacetylene composed of [-SiR_2_-C≡C-Ar-]. The results showed that the T_d5_ and Y_c_ in nitrogen at 1000 °C could reach 715 °C and 93%, respectively. 

The organic-inorganic hybrid polymer material is also a considerable option for better properties in thermal protection systems [18,19]. Therein, ceramifiable polymer composites are paid more attention in the field of thermal protection system due to their advantages of low heat conduction, excellent mechanical properties and ablation resistance [20,21]. The silicon, nitrogen and zirconium elements have been used as ceramifiable precursors. Wang M. C. et al. [22] prepared a novel liquid poly(silylacetylene)siloxane resin, as a novel precursor of silicon carbide and silicon oxycarbide ceramics. The results showed that the T_d5_ and Y_c_ in nitrogen at 900 °C were above 400 °C and 80%, respectively. Additionally, silicon oxycarbide ceramic was obtained at a yield of >75% by pressureless pyrolysis at 900–1200 °C, and silicon carbide ceramic was obtained at 1500 °C. 

There are some studies about the polymer materials modified by zirconium element, which is commonly used in the field of ceramics, because zirconium dioxide is an excellent toughening ceramic material [23]. In our previous study [24], silicone rubber composites filled with zirconium silicide were prepared by physical blending. The results showed that the T_d5_ and Y_c_ at 1000 °C in air were 399.7 °C and 45.48%, respectively. When heated to a high temperature, the zirconium would be oxidized to produce the ZrO_2_ ceramic phase and dispersed evenly on the surface of materials, and prevent further heat oxidation and decomposition. Introducing zirconium-containing filler into the resin matrix was a good way to improve heat resistance, but it also increased the viscosity, which definitely caused trouble for the preparation of FRP.

In order to further improve the thermal property of PAA based FRP for the thermal protection field, the introduction of the zirconium element into arylacetylene will be promising. In this paper, zirconium modified arylacetylene resin was prepared by a two-step synthesis. The molecular chemical structures were investigated by Fourier transform infrared spectroscopy (FT-IR). The curing kinetics were studied by differential scanning calorimetry (DSC). The thermal stability was researched using a thermogravimetric analyzer (TGA) to identify the effect of zirconium on the silicon-containing arylacetylene. The ceramifiable mechanism was studied by the X-ray diffraction (XRD), X-ray fluorescence (XRF) and scanning electron microscope (SEM).

## 2. Experimental

### 2.1. Materials

The solution of hydrogen chloride in ethyl acetate (1 mol/L) was obtained from Changtai Pharmaceutical Co. Ltd (Zhenjiang, China). The anhydrous ether (AR, purity 99.7%), ammonium chloride (AR, purity 99.5%) and tetrahydrofuran (AR, purity 99.0%) were purchased from Sinopharm Chemical Reagent Co. Ltd (Shanghai, China). The zirconium tetrachloride (AR, 99.9%), m-amino arylacetylene (purity 99.0%) and trimethylchlorosilane (GC, purity 99.0%) were provided by Aladdin Industrial Co. Ltd (Shanghai, China). All of the chemicals were used as received without further purification.

### 2.2. Synthesis of silane terminated m-amino arylacetylene (SAA)

**Scheme 1:**
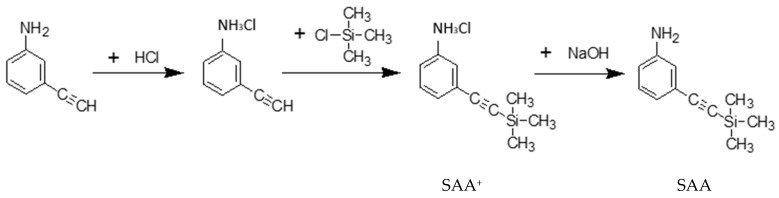
Synthesis of silane terminated m-amino arylacetylene (SAA).

The synthesis of silane terminated m-amino arylacetylene (SAA) is shown in Scheme 1. The m-amino arylacetylene (0.2 mol, 23.4g) was dropwise added into the solution of hydrogen chloride dissolved in ethyl acetate (200 mL). With the reaction processing, the white crystals (m-ammonium arylacetylene) precipitated in the bottom of the round-bottom flask. Then, the white crystals were washed twice by ethyl acetate and dried in a vacuum drying oven at 80 °C for 4 h, to obtain m-ammonium arylacetylene. The whole reaction process of the synthesis of SAA was protected by nitrogen atmosphere and controlled at 0 °C by ice-water bath. The Anhydrous ether (40mL) and tetrahydrofuran (40mL) were added into a 250 mL round-bottom flask. Then the m-ammonium arylacetylene (4.72g, 0.04 mol) was added into the flask. The reaction was maintained at 0 °C and stirred for 20 min. After that, trimethylchlorosilane was dropwise added into the solution. The solution was also maintained at 0 °C and stirred for 60 min. After the reaction, the reaction mixture was filtered, and brown powder solid (silane terminated m-ammonium arylacetylene, abbreviated as SAA^+^) was obtained on the filter paper. Then, the brown powder solid was dissolved in distilled water and titrated by NaOH solution (1 mol/L), until the pH value of the solution was 7. After the reaction, the reaction mixture was filtered, and brown powder solid (silane terminated m-amino arylacetylene, abbreviated as SAA) was obtained. At last, the brown powder was put into the vacuum drying oven at 40 °C for 24 h to get rid of the solvent and obtain the SAA.

### 2.3. Synthesis of zirconium modified arylacetylene (ZAA) Resin

**Scheme 2:**
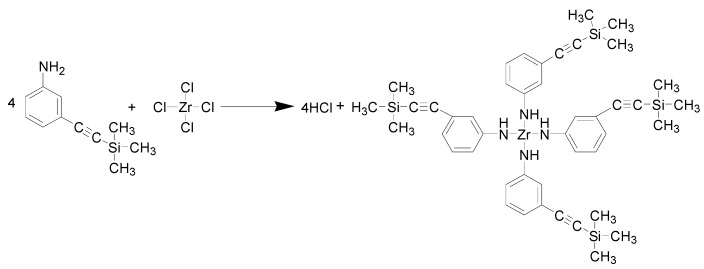
Synthesis of zirconium modified arylacetylene (ZAA) resin.

The synthesis of zirconium modified arylacetylene (ZAA) resin is shown in Scheme 2. The whole reaction process of the synthesis of ZAA was protected by a nitrogen atmosphere at room temperature. Anhydrous ether (100 mL) and prepared SAA (7.56 g) were added into a 250 mL round-bottom flask. The solution of zirconium tetrachloride (2.33 g, 0.01 mol) dissolved in tetrahydrofuran (40 mL), was dropwise added into a 250 mL round-bottom flask and stirred for 20 h to react. After the reaction, the solution was filtered by a separatory funnel to obtain the upper oil phase. The oil phase was washed by an ammonium chloride saturated solution, until the pH value of the filtrate was near 7. Rotary evaporator and vacuum drying were utilized to get rid of solvent, to obtain the brown solid of ZAA. 

### 2.4. Preparation of poly(zirconium modified arylacetylene) (PZAA) and poly(silane terminated m-amino arylacetylene) (PSAA)

The SAA and ZAA resin were grinded by a planetary ball mill to get a small particle sample. The grinding balls were made of ZrO_2_, and the diameter of each ball was 2 mm. The samples were grinded for 40 min at 360 rpm. The mold was preheated in an oven at 130 °C for 0.5h. The release agent was evenly sprayed on the surface of the mold to facilitate demoulding. SAA and ZAA (12 g) were respectively weighed and put into the mold. The SAA and ZAA were cured in an air circulating oven by the following steps: 130 °C (1 h), 150 °C (2 h), 180 °C (4 h) and 225 °C (2 h).

### 2.5. Instruments

Fourier transform infrared spectroscopy (FT-IR) was recorded on KBr pellets from 4000 to 400 cm^−1^ by a Nicolet Nexus IR Spectra, made in Madison, WI, USA. 

A viscosity-temperature relationship was performed from 70 °C to 160 °C at a heating rate of 5 °C/min, using a TA instrument (AR-2000 rheometer, made in TA Instruments, Newcastle, UK), with a 25 mm diameter.

The differential scanning calorimetry (DSC) studies were conducted on the blends in hermetically sealed pans. The heating and cooling experiments were performed at 5, 10, 15, 20 °C/min, with Perkin Elmer DSC7, made in Waltham, MA, USA. The sample (10 mg) was sealed under nitrogen in aluminum pans. Temperature ramping DSC studies during curing were performed from 50 to 300 °C. 

The thermal stability of samples was measured with a Mettler SDTA 851 thermogravimetric analyzer (TGA, made in Zurich, Switzerland) at a heating rate of 10 °C/min.

The morphology of the blends was studied by examining the fracture surfaces, using a German Leica polarizing microscope DM4500P (made in Leica, Weztler, Germany. The specimens were freeze-fractured using liquid nitrogen and then sputter-coated with silver and mounted on a carbon tape prior to the scanning electron microscope (SEM) examination. 

An X-ray diffraction (XRD) analysis was conducted to analyze the chemical component of the product after ablation at 1000 °C. The samples were measured by monitoring the diffraction angle 2 θ from 10° to 80° on a D8Wance X-ray powder diffractometer of AXS Corporation made in Brock, Karlsruhe, Germany.

X-Ray Fluorescence (XRF) Spectroscopy was recorded to analyze the element content in the product after ablation at 1000 °C. The samples were measured by the AXIOS XRF spectrum instrument (made in Almelo, Holland) with a Cu target, K_β_ filter of Ni, voltage 35–45 kV, current 30–40 mA.

## 3. Results and Discussion

### 3.1. FTIR Analysis

**Figure 1:**
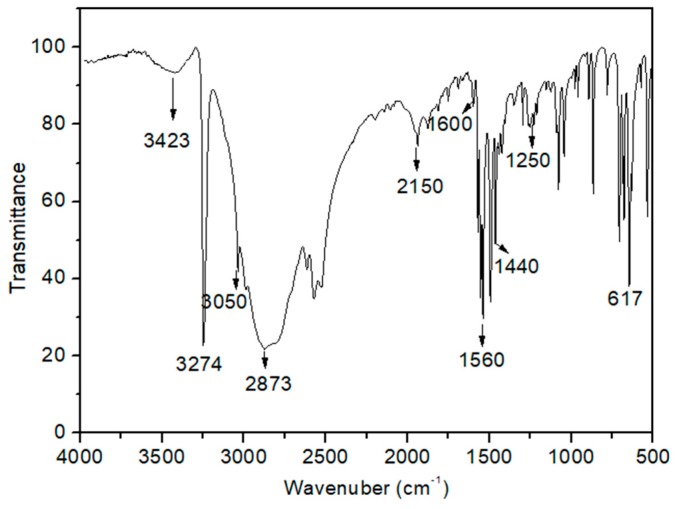
The FTIR spectrum of SAA^+^.

The molecular structure of SAA^+^ was characterized with FTIR, as shown in Figure 1. In the FTIR spectrum, the small and broad absorption peak located at 3423 cm^−1^ belongs to N-H of Ar-NH_2_, which meant the additive amount of m-amino arylacetylene was in excess than hydrogen chloride. The absorption peak at 3274 cm^−1^ is due to the C-H of methyl stretching vibration. The absorption peaks at 3050 cm^−1^ characterize the C-H stretching vibration in benzene ring structure. The wide absorption peak around 2873 cm^−1^ characterizes the N-H of -NH_3_^+^ stretching vibration. The absorption peak around 2150 cm^−1^ belongs to -C≡C-Si stretching vibration. The absorption peak 1560 cm^−1^ is due to the Ar-N stretching vibration and bending vibration. The absorption peaks at 1440 cm^−1^ and 1600 cm^−1^ correspond to C-C stretching vibration of benzene ring skeleton. The absorption peak at 1250 cm^−1^ is due to the symmetrical deformation vibration of Si-CH_3_. The absorption peak at 616 cm^−1^ is the stretching vibration of the Si-C bond. Based on the above FTIR absorption peaks analysis, the characteristic functional groups in the structure of the SAA could be determined, and the results showed that the molecular structure of the SAA accorded with the structure, as shown in Scheme 1.

**Figure 2:**
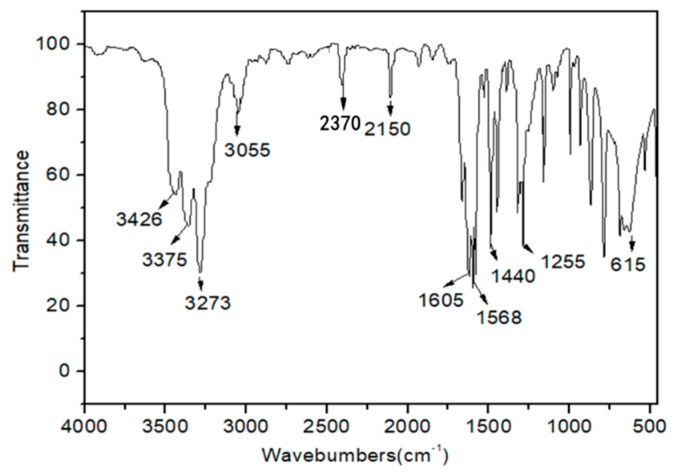
The FTIR spectrum of ZAA.

The molecular structure of ZAA was characterized with FTIR, as shown in Figure 2. In the FTIR spectrum, the broad absorption peaks at 3426 cm^−1^ and 3375 cm^−1^ are respectively due to the N-H of Ar-NH_2_ and Ar-NH-, which meant the Ar-NH_2_ of ZAA was not totally consumed. The absorption peak at 3273 cm^−1^ is due to the C-H of methyl stretching vibration. The absorption peaks at 3055 cm^−1^ characterizes the C-H stretching vibration in benzene ring structure. The absorption peak at 2150 cm^−1^ belongs to -C≡C-Si stretching vibration. The absorption peak 1568 cm^−1^ is due to the Ar-N stretching vibration and bending vibration. The absorption peaks at 1440 cm^−1^ and 1605 cm^−1^ correspond to the C-C stretching vibration of benzene ring skeleton. The absorption peak at 1255 cm^−1^ is due to the symmetrical deformation vibration of Si-CH_3_. Compared with SAA, there is a new absorption peak at 2370 cm^−1^ in FTIR spectrum of ZAA, which is probably due to the Zr-N group. Based on the above FTIR absorption peaks analysis, the characteristic groups in the structure of the ZAA could be determined, and the results showed that the molecular structure of the ZAA accorded with the structure in Scheme 2.

### 3.2. Rheological Analysis

**Figure 3:**
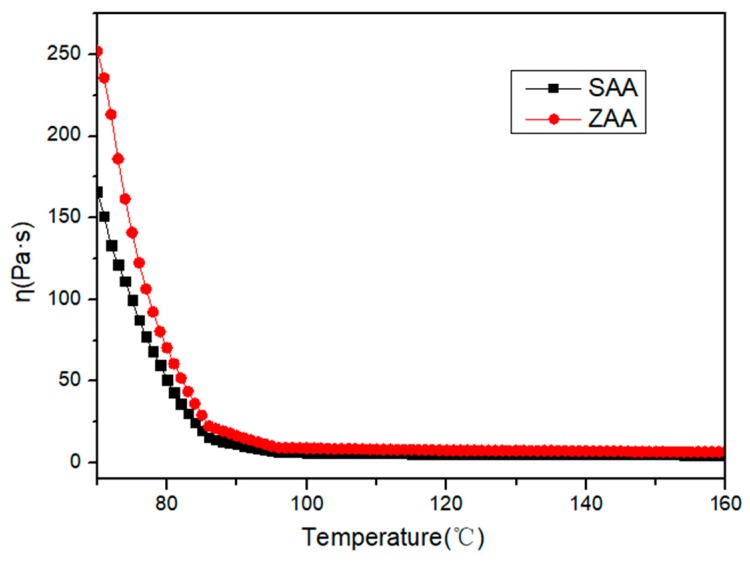
The viscosity-temperature relation of SAA and ZAA.

The viscosity-temperature relations of SAA and ZAA were measured at a heating rate of 5 °C/min. As shown in Figure 3, the viscosities of both resins decreased dramatically, with temperature increasing from 70 °C to 85 °C, and decreased gently, with temperature increasing from 85 °C to 95 °C, and finally became steady with temperature increasing from 95 °C to 160 °C. Compared with SAA, the ZAA had an extremely high viscosity above 250 Pa·s at 70 °C. Nevertheless, when the temperature was above 120 °C, the viscosity of ZAA was about 6.5 Pa·s, which was approximate to SAA (4.9 Pa·s). Therefore, the results showed that the ZAA resin had as wide processing window as SAA.

### 3.3. DSC Analysis

**Figure 4:**
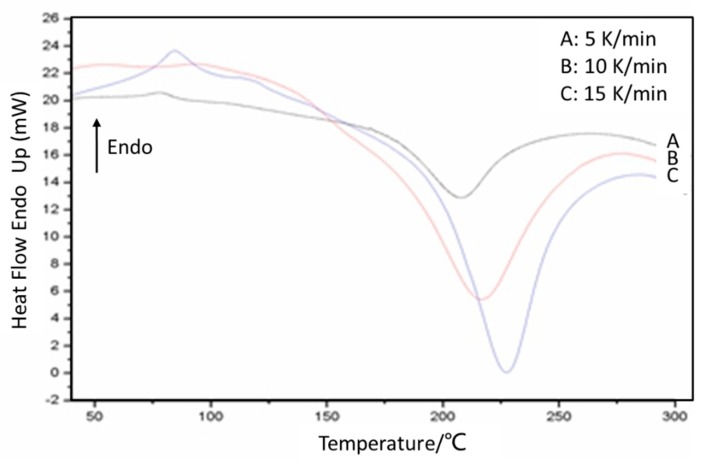
ZAA curing exothermic curve under different heating rate.

Figure 4 shows the DSC curing exothermic curve of ZAA at the heating rate of 5 °C/min, 10 °C/min and 15 °C/min, respectively. It could be seen that the exothermic rate increased with the heating rate increasing, and the peak temperature also drifted towards a higher temperature due to the thermal hysteresis effect. There is a small endothermic peak around 50–70 °C in the curves of heating rates of 5 and 15 °C/min, which was caused by the volatilization of some small molecule solvents. The initial temperature, peak temperature and end temperature of curing exotherm at different heating rates are tabulated in Table 1. As showed in Table 1, the characteristic temperatures at 5 °C/min heating rates were lowest, compared with others. With the characteristic temperatures at the different heating rate identified, the characteristic temperatures of heating rate at 0 °C/min can be obtained according to the extrapolation method. The results are showed in Table 1.

The Kissinger and the Ozawa methods were commonly used to deal with DSC curve data for all parameters of the curing reaction kinetics. According to the Kissinger method, it is assumed that the curing reaction rate maximizes at the peak temperature in DSC curve. Meanwhile, the Ozawa method assumes that the reaction conversion rate is a constant at the peak temperature. Further transformed, the Kissinger and the Ozawa Equation are illustrated in Equations (1) and (2), respectively.

(1) $$\ln\left(\frac{\beta }{T_{p}{}^{2}}\right)=\ln\left(\frac{Q_{p}AR}{E_{a}}\right)-\frac{E_{a}}{RT_{p}}$$

(2) $$\ln\beta =-\frac{1.052E_{a}}{RT_{p}}+C$$

where β is heating rate (K/min); Tp is curing peak temperature (K); Ea is activation energy (kJ/mol); R is the ideal gas constant (8.314 J/mol·K); C is constant; Qp is exothermic enthalpy; and A is the prefactor. 

**Figure 5:**
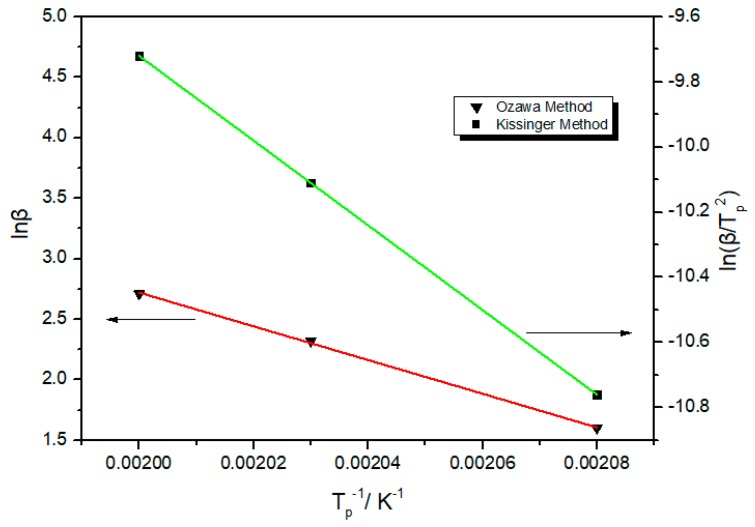
Kissinger method and Ozawa method fitting figure of ZAA.

The Kissinger method and Ozawa method fitting figures of ZAA are showed in Figure 5. The apparent activation energy of ZAA curing reaction could be calculated to obtain a value of 103.86 kJ/mol in Kissinger method and 106.46 kJ/mol in Ozawa method.

### 3.4. Dielectric Constant

**Figure 6:**
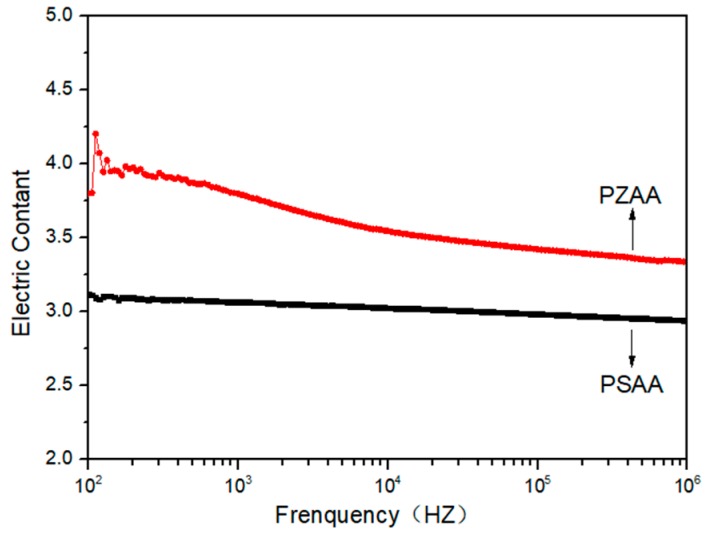
Frequency dependences of the dielectric constant of PSAA and PZAA.

Figure 6 shows the dielectric constant versus frequency from 10^2^ to 10^6^ Hz curves of PSAA and PZAA. The dielectric constant displayed little change with the increasing of frequency, which indicated the PSAA and PZAA possessed stable dielectric property. The dielectric constant at 1 MHz of PSAA and PZAA are 3.0 and 3.4, respectively. This was because that the PSAA and PZAA were composed of an aromatic and cross-linked network of cured acetenyl with low dielectric constant. The dielectric constant of PZAA was higher than PSAA, because the zirconium had a high dielectric constant of 33 and the introduction of zirconium decreased the crosslinking density.

### 3.5. TGA Analysis 

**Figure 7:**
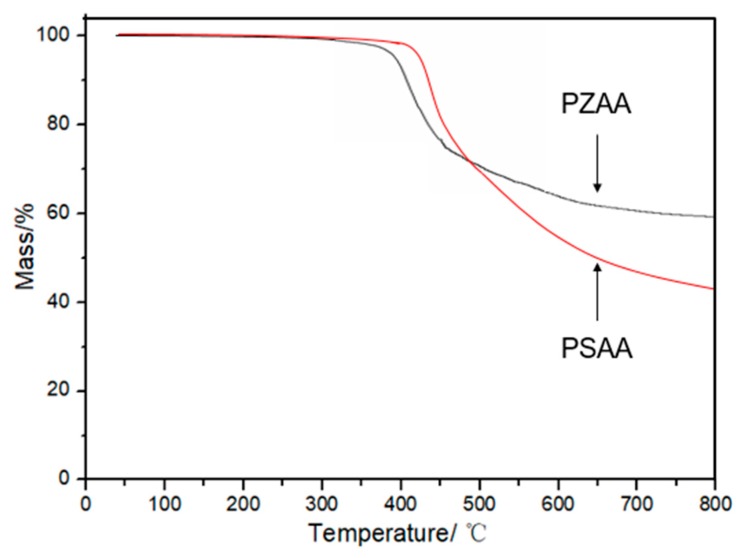
TGA curves of PSAA and PZAA in air.

A thermogravimetric analysis (TGA) in air was conducted to provide the thermal property information of PSAA and PZAA resins. The TGA curves in air atmosphere are showed in Figure 7, and the characteristic parameters of the thermal decomposition are listed in Table 2. The two cured arylacetylene exhibited high thermal stability, of which T_d5_s and Y_c_s at 800 °C were above 400 °C and 40%, respectively. This indicated that polyarylacetylene possessed good thermal stability, because acetylene group could form a highly cross-linked and thermally stable network structure. The T_d5_ of PSAA is higher than PZAA by 22.5 °C, which indicated that the introduction of zirconium into PZAA decreased the thermal stability. This is because the star-shaped arylacetylene resin made it difficult for acetenyl to react with each other and there were decreases in the crosslinking density of PZAA. With temperature increasing further, the mass of cured resins changed sharply, and the polyarylacetylene decomposed violently. It is obvious that the mass of PSAA decreased sharply than PZAA and both resins shared one Y_c_ of 72.3% at 485 °C. When the temperature was 700 °C, the decomposition of PZAA was basically over, but the decomposition of PSAA still processed. The Y_c_ at 800 °C of PZAA and PSAA are 61.4% and 41.2%, respectively. Therefore, the introduction of zirconium into PZAA had little negative effect on T_d5_, but improved the Y_c_ at 800 °C greatly. 

### 3.6. XRD Analysis 

**Figure 8:**
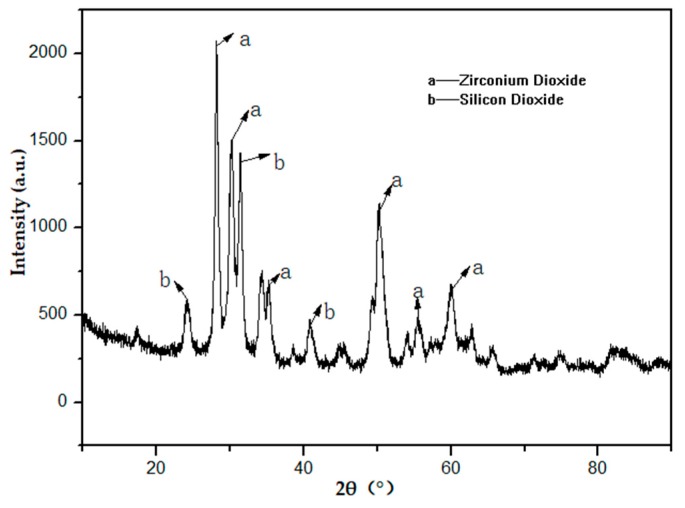
XRD curves of PZAA after ablation at 1000 °C in air.

In order to further characterize the components of the PZAA residues after ablation, an XRD analysis was performed and the peak spectra is showed in Figure 8. As showed in the figure, the whole XRD diffraction peak spectrum showed that there was no glass phase in the sample, but only a crystalline phase structure. The existing crystal phase peaks could be divided into two main types. The strong diffraction peaks at 28.3°, 30.2°, 35.4°, 50.2°, 55.7° and 60.0° are due to zirconium dioxide (ZrO_2_), which could be referred to as the standard CARDS with codes 65-0461 (zirconia). Further analysis showed that the strong diffraction peak at 30.2° could correspond to a 111 crystal plane in ZrO_2_ structure. The diffraction peaks at 50.2° and 60.0° corresponded to the diffraction peaks on the 202 and 131 crystal face of ZrO_2_, indicating that PZAA mainly formed a ZrO_2_ crystal phase after sintering at 1000 °C. The strong diffraction peaks at 24.4°, 31.67° and 40.9° are the diffraction peaks of silicon dioxide (SiO_2_), and the reference code is 46-1045. The results showed that the PZAA contained a silica phase in the ablated residues, and formed a SiO_2_ crystal phase after ablation. The presence of ZrO_2_ and SiO_2_ indicated that the resin can form a ceramic structure after ablation in the oxygen-containing atmosphere, and the thermal insulation and high temperature resistance can protect the internal structure of the resin.

In order to figure out the quantitative analysis of PZAA after ablation, XRF spectroscopy was conducted to obtain the content of components. As showed in Table 3, the loss on ablation of PZAA is 54.2%. The contents of SiO_2_ and ZrO_2_ are 15.3% and 12.4% after ablation, respectively. According to the structure analysis of the resin, the loss components were mainly C, H, N elements, while the remaining elements were mainly Zr and Si, with a small amount of elements, including Al, Fe, Ca and Cl, which further indicated that the purity of the resin was very high. As we knew, the contents of SiO_2_ and ZrO_2_ are 15.3% and 12.4%, so the atomic mole ratio of Si:Zr was 2.3, which meant the ZAA was principally composed of dibasic and tribasic Zr. The results also showed that the remaining SiO_2_ and ZrO_2_ were principally attributed to the high Y_c_ of PZAA, while there was still 13% C, H and N in the structure. 

### 3.7. SEM Analysis 

**Figure 9:**
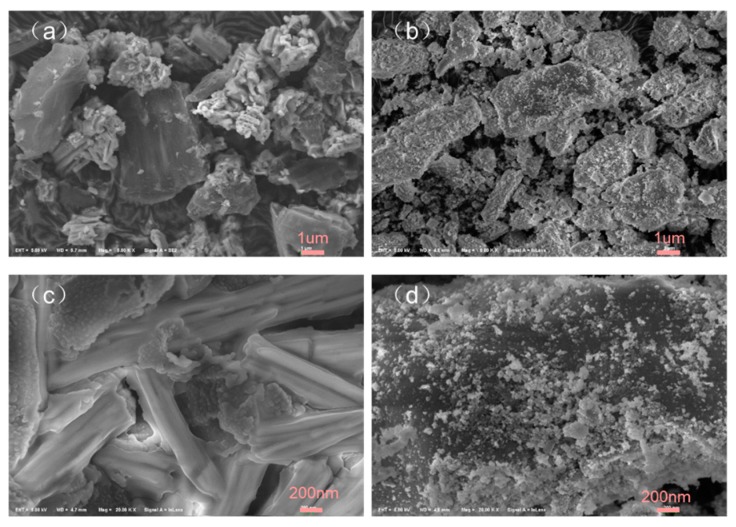
SEM spectrum of PZAA before ablation at 1000 °C (**a**,**c**); after ablation at 1000 °C (**b**,**d**).

Figure 9 shows the comparison in the microstructure of PZAA before and after ablation at 1000 °C. Through observation and analysis, it could be found that the resin in Figure 9a agglomerated together to form a state of similar size and uniform distribution, with close distances between blocks. After ablation, the residues in the resin were lumpy, and the lumps were distributed evenly with large differences in the size, which was showed in Figure 9b. The results also showed a loose structure in the pyrolytic structure, from which lots of gaseous substances ware released due to the degradation of polyarylacetylene. In Figure 9c, after the increase of the magnification factor, it can be seen that there is much villous material on the surface of the block which are distributed crosswise. In Figure 9d, the surface of residues is covered with a dense and rigid structure, which consisted of SiO_2_ and ZrO_2_ and could resist the erosion of high-speed airflow. Through a comparative analysis, the microstructure changes of PZAA before and after ablation could be observed in the SEM spectrums. After ablation, the lumps on surface of PZAA became rough and distributed, with larger differences in the size than the PZAA before ablation. It could be concluded that the surface of PZAA after ablation was packeted with a ceramic phase composed of SiO_2_ and ZrO_2_. The formation of SiO_2_ and ZrO_2_ ceramic phase on the matrix surface could result in the reduction of heat and oxygen gas transferring to the internal matrix. Therefore, the introduction of Zr into arylacetylene greatly improved the densification of ceramic products during ablation, and improved the heat resistant property.

## 4. Conclusions

In the current study, a high-performance modified zirconium and silicon containing arylacetylene resin were successfully synthesized. The FTIR analysis characterized and confirmed the products and their molecular structures. The viscosity of zirconium modified arylacetylene (ZAA) was about 6.5 Pa·s when temperature was above 120 °C, which showed that the ZAA resin had a wide processing window. The DSC analysis showed that the ZAA had a low curing temperature, and its apparent activation energy was 103.86 kJ/mol in Kissinger method and 106.46 kJ/mol in Ozawa method. The dielectric constant at 1 MHz of PZAA was 3.4, which indicated that poly(zirconium modified arylacetylene) (PZAA) possessed a low dielectric constant. The TG analysis showed that the temperatures of a weight loss of 5% (T_d5_) of poly(silane terminated m-amino arylacetylene) (T_d5_ of 435.2 °C, abbreviated as PSAA) was higher than PZAA (T_d5_ of 407.5 °C) by 22.5 °C, because the star-shaped arylacetylene resin made it difficult for acetenyl to react with each other and decreases of crosslinking density of PZAA. The Y_c_ at 800 °C of PZAA and PSAA are 61.4% and 41.2%, respectively. Therefore, the introduction of zirconium into PZAA had a bad effect on T_d5_, but improved the Y_c_ at 800 °C greatly. The XRD results showed the presence of SiO_2_ and ZrO_2_ in the molecular structure after ablation. The XRF results showed the contents of SiO_2_ and ZrO_2_ in PZAA residual, after ablation were respectively 15.3% and 12.4%, which meant that ZAA was principally composed of dibasic and tribasic Zr and the remaining SiO_2_ and ZrO_2_ were principally attributed to the high Y_c_ of PZAA. The SEM showed that the surface of PZAA after ablation had been packeted with a ceramic phase composed of ZrO_2_. The formation of a ZrO_2_ ceramic phase on the matrix surface could result in the reduction of heat and oxygen gas transferring to the internal matrix. Therefore, the introduction of Zr into arylacetylene greatly improved the densification of ceramic products during ablation, and improved the heat resistant property. According to the above results, as a high heat resistant resin, the zirconium modified arylacetylene will be promising in the preparation of fiber reinforced composites for thermal protection systems.

## Figures and Tables

**Table 1:** The characteristic temperatures at various heating rates of ZAA.

Heating Rate (°C/min)	Onset (°C)	Peak (°C)	End (°C)
5	179.4	207.4	232.1
10	190.2	219.2	245.3
15	198.3	227.2	251.4
0	170.4	198.3	224.6

**Table 2:** TGA analysis results of BA and BSA in air.

Resin	In Air	
	T_d5_/°C	Y_c_ at 800 °C
PSAA	435.2	41.2%
PZAA	412.7	61.4%

**Table 3:** XRF result of PZAA.

Components	Weight Contents/%	Components	Weight Contents/%
Al_2_O_3_	0.3	Fe_2_O_3_	0.1
SiO_2_	15.3	Cl	1.1
CaO	1.0	ZrO_2_	12.4
Loss on ablation	54.2	——	——

## References

[1] Kumar S.R., Dhanasekaran J., Mohan S.K. (2015). Epoxy benzoxazine based ternary systems of improved thermo-mechanical behavior for structural composite applications. RSC Adv..

[2] Sandomierski M., Strzemiecka B., Koczorowski W., Barczewski M., Kasperkowiak M., Pokora M., Borek B., Chehimi M.M., Voelkel A. (2018). Mechanically robust and thermally stable abrasive tools from phenolic resins reinforced with diazonium-modified zeolites. Polym. Compos..

[3] Joseph S., Sreekala M.S., Thomas S. (2008). Effect of chemical modifications on the thermal stability and degradation of banana fiber and banana fiber-reinforced phenol formaldehyde composites. J. Appl. Polym. Sci..

[4] Ogunsona E.O., Codou A., Misra M., Mohanty A.K. (2018). Thermally Stable Pyrolytic Biocarbon as an Effective and Sustainable Reinforcing Filler for Polyamide Bio-composites Fabrication. J. Polym. Environ..

[5] Wang S., Li M., Gu Y., Zhang Z. (2010). Experimental Study on Crack Defects Formation in Polyarylacetylene Composites and Modification Improvement of Resin. J. Compos. Mater..

[6] Jiang Z.X., Liu L., Huang Y.D., Ren H. (2009). Influence of coupling agent chain lengths on interfacial performances of carbon fiber and polyarylacetylene resin composites. Surf. Interface Anal..

[7] Zhang J. (2007). Fiber reinforced silicon-containing arylacetylene resin composites. Express Polym. Lett..

[8] Thompson C.M., Hergenrother P.M. (2002). Aryl Ethynyl Terminated Imide Oligomers and Their Cured Polymers. Macromolecules.

[9] Chu Q., Zhang J., Xu Z., Ding M. (2000). Study on a novel polyimide precursor prepared by a modified polymerization of monomeric reactants (MPMR) procedure. Macromol. Chem. Phys..

[10] Hergenrother P., Connell J., Smith J. (2000). Phenylethynyl containing imide oligomers. Polymer.

[11] Homrighausen C.L., Keller T.M. (2001). High-temperature elastomers from silarylene-siloxane-diacetylene linear polymers. J. Polym. Sci. Part A Polym. Chem..

[12] Ohshita J., Iida T., Izumi Y., Kunai A. (2006). Synthesis of Poly[(cyanophenyl)silylene-p-phenylene]s as Patternable Ceramics Precursors. J. Ceram. Soc. Jpn..

[13] Ohshita J., Shinpo A., Kunai A. (1999). Synthesis of Poly[bis(ethynylphenyl)silylene] phenylene}s with Highly Heat-Resistant Properties. Macromolecules.

[14] Kuroki S., Okita K., Kakigano T., Ishikawa J.-I., Itoh M. (1998). Thermosetting Mechanism Study of Poly[(phenylsilylene)ethynylene-1,3-phenyleneethynylene] by Solid-State NMR Spectroscopy and Computational Chemistry. Macromolecules.

[15] Zhang J., Huang J., Wang C., Huang F., Du L. (2009). A New Silicon-Containing Arylacetylene Resin With Amine Groups as Precursor to Si−C−N Ceramic. J. Macromol. Sci. Part B.

[16] Li Q., Zhou Y., Hang X., Deng S., Huang F., Du L., Li Z. (2008). Synthesis and characterization of a novel arylacetylene oligomer containing POSS units in main chains. Eur. Polym. J..

[17] Guo K., Li P., Zhu Y., Wang F., Qi H. (2016). Thermal curing and degradation behaviour of silicon-containing arylacetylene resins. Polym. Degrad. Stab..

[18] Huang C.-F., Kuo S.-W., Lin F.-J., Huang W.-J., Wang C.-F., Chen W.-Y., Chang F.-C. (2006). Influence of PMMA-Chain-End Tethered Polyhedral Oligomeric Silsesquioxanes on the Miscibility and Specific Interaction with Phenolic Blends. Macromolecules.

[19] Matejka L., Strachota A., Plestil J., Whelan P., Steinhart M., Slouf M. (2004). Epoxy networks reinforced with polyhedral oligomeric silsesquioxanes (POSS). Structure Morphology. Macromolecules.

[20] Ding J., Yang T., Huang Z., Qin Y., Wang Y. (2018). Thermal stability and ablation resistance, and ablation mechanism of carbon–phenolic composites with different zirconium silicide particle loadings. Compos. Part B Eng..

[21] Ding J., Huang Z.X., Qin Y., Shi M.X., Huang C., Mao J.W. (2015). Improved ablation resistance of carbon-phenolic composites by introducing zirconium suicide particles. Compos. Part B Eng..

[22] Wang M., Yang L., Yu C., Xu C. (2012). (Charles) Liquid poly(silylacetylene)siloxane resin as a novel precursor of silicon carbide and silicon oxycarbide ceramics. Ceram. Int..

[23] Chen Y., Hong C., Chen P. (2013). The effects of zirconium diboride particles on the ablation performance of carbon–phenolic composites under an oxyacetylene flame. RSC Adv..

[24] Song J., Huang Z., Qin Y., Wang H., Shi M. (2020). Effects of Zirconium Silicide on the Vulcanization, Mechanical and Ablation Resistance Properties of Ceramifiable Silicone Rubber Composites. Polymers.

